# Evaluation of neurofibromatosis type 1 progression using a nationwide registry of patients who submitted claims for medical expense subsidies in Japan between 2008 and 2012

**DOI:** 10.1186/s13023-019-1148-8

**Published:** 2019-07-05

**Authors:** Takashi Yamauchi, Machi Suka, Chikako Nishigori, Hiroyuki Yanagisawa

**Affiliations:** 1 Department of Public Health and Environmental Medicine, The Jikei University School of Medicine, 3-25-8 Nishi-shimbashi, Minato-ku, Tokyo, 105-8461 Japan; 2 Graduate School of Medicine, Kobe University, Kobe, Japan

**Keywords:** Intractable rare disease, Neurofibromatosis type 1, Progression, Follow-up, National registry, Medical expense subsidies, Japan

## Abstract

**Background:**

No study to date has followed disease progression in patients with neurofibromatosis type 1 (NF1), including the incidence of various manifestations, using a national registry. Here we examined the state of NF1 progression using a nationwide registry of patients who submitted claims to receive medical expense subsidies for NF1 in Japan over a five-year period. A total of 342 eligible patients (194 females and 148 males) with NF1 who newly submitted claims for medical expense subsidies in Japan in 2008 were followed until 2012.

**Results:**

More than half of the patients were classified as Stage 5 in 2008. Of the eligible patients, 205 (60%) submitted claims to renew the subsidies between 2009 and 2012. During the study period, NF1 stage progressed in 30 patients, yielding an overall stage progression rate of 19% and progression incidence rate per 100 person-years of 12.2. Both stage progression rate and progression incidence rate were the highest in the 0–19 year age group at the time of registration and, as compared to other age groups, progression of neurological and bone manifestations was more prevalent in this age group.

**Conclusions:**

The progression of neurological and bone manifestations was more prevalent in the 0–19 year age group compared to other age groups. The registry we used in the present study is useful for understanding the characteristics of patients with uncommon conditions, such as NF1. Our findings also highlight the feasibility of conducting quality research using registries of patients with rare diseases, such as NF1, that were not designed specifically for scientific research.

**Electronic supplementary material:**

The online version of this article (10.1186/s13023-019-1148-8) contains supplementary material, which is available to authorized users.

## Introduction

There are currently more than 6000 identified intractable rare diseases worldwide [1, 2]. Neurofibromatosis type 1 (NF1) is a relatively common inherited disorder that affects approximately one in 2500 to one in 3000 people worldwide, irrespective of sex or ethnic background [3, 4]. Many manifestations of NF1 affect the skin, the nervous system, and bones [5–7].

While cross-sectional epidemiological studies of NF1 have been conducted in western countries using large registries [8–10], only a few studies have examined the characteristics, including the natural course, of NF1 using population-based registries with follow-up data [11–13]. However, these studies specifically focused on the incidence of cancer in patients with NF1. To our knowledge, no study has followed the clinical stage of patients with NF1, including the incidence of various manifestations apart from cancer, using a national registry.

Japan has implemented globally unprecedented and comprehensive measures against intractable rare diseases (*nanbyou* in Japanese) [1, 14]. Patients who meet certain severity grades of these diseases, including NF1, become eligible for medical expense subsidies because these diseases have established diagnostic criteria, but are difficult to investigate or develop treatments for unless public funds are available to cover expenses. Certain rare diseases, including NF1, have been referred to as “designated intractable diseases” in Japan (331 diseases as of April 2018). While intractable diseases must fulfil four criteria (i.e., unknown pathogenic mechanism, lack of established treatment, rarity, and necessity of long-term treatment), designated intractable diseases must meet two additional criteria: the number of patients in Japan has not reached a certain threshold (approximately 0.1% of the population), and the existence of objective diagnostic criteria (or equivalent).

Thus, the present exploratory study aimed to examine the state of NF1 progression in Japan using a national registry of all patients who submitted claims for medical expense subsidies for NF1 over a five-year period. A better understanding of the state of progression of NF1 using longitudinal national registry data may contribute to the welfare of patients with NF1, including career support, the development of evidence-based clinical guidelines, and improvement of the national health care system for intractable rare diseases.

## Methods

### Study population and data source

We used a database containing information on all patients who submitted claims for medical expense subsidies for NF1 in Japan between fiscal years 2008 and 2012. The number of NF1 patient registration in Japan during this 5-year period remained more than 300. This might be partially due to the online registry system developed by the Ministry of Health, Labour and Welfare (MHLW) aimed at standardising the registration process for medical expense subsidies. We chose this 5-year period as the study period in order to limit the effect of potential selection bias.

The Intractable/Rare Disease Control Division within the MHLW provided the database following approval of the research protocol. The Division removed all identifiable information, such as names and addresses, from the database and coded a unique identifiable number (ID number) for each patient. The ID number corresponded to seven digits that were not associated with any identifiable personal information. Data linkage of the annual datasets (i.e., dataset for initial claims in 2008 and those for renewal claims from 2009 to 2012) was conducted by ID number, age, and sex.

**Fig. 1:**
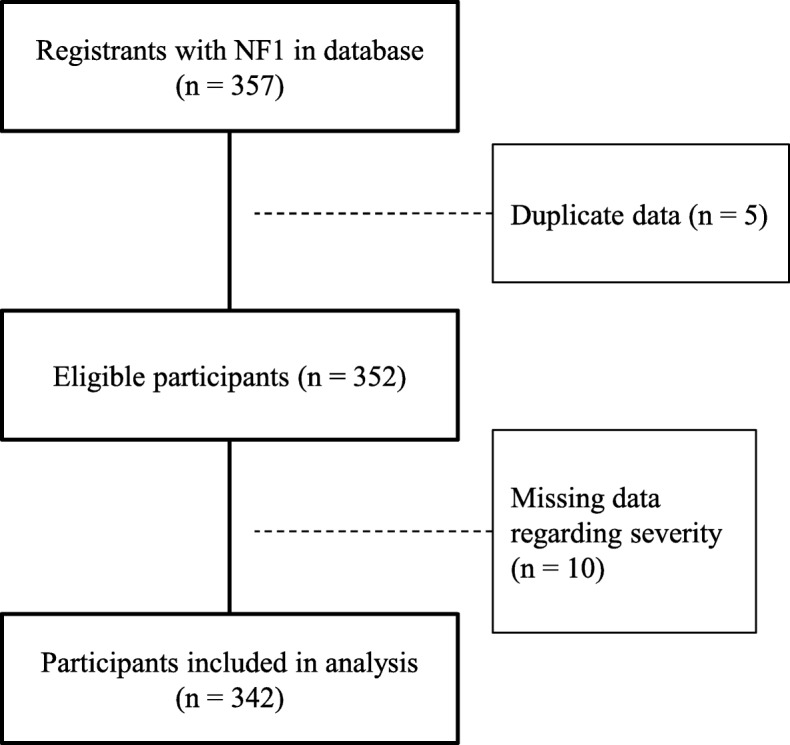
Participant selection

To receive medical expense subsidies, applicants must submit a claim to the prefectural government via a local public health centre. If the claim is approved and applicants wish to continue receiving the subsidy, they must submit a renewal application on an annual basis. In fiscal year 2008, 357 patients newly (i.e., for the first time) submitted claims to receive medical expense subsidies for NF1 (Fig. 1).

In Japan, diagnostic criteria and a disease severity classification for NF1 have been established in order to standardise the criteria for medical expense subsidies. Physicians fill out a medical certificate (“clinical personal record”) according to assessment results based on diagnostic criteria and disease severity. This clinical personal record is required for the applicant to receive subsidy payments for intractable rare diseases, and the MHLW has accumulated this information in the registry. This overall process may reduce the influence of information bias on the present study.

The database we used included the following information: (1) age at registration, (2) severity of dermatological manifestations (D1: pigmented macules and few neurofibromas, D2: pigmented macules and many neurofibromas, D3: numerous neurofibromas (> 1000), D4: functional impairment or severe physical distress due to plexiform neurofibromas, or malignant peripheral nerve sheath tumour), neurological manifestations (N0: no neurological symptoms, N1: neurologic symptoms (e.g., paralysis or pain), N2: severe/progressive neurological symptoms), and bone manifestations (B0: no bone lesions, B1: mild/moderate bone lesions (e.g., deformity in spine that does not require surgical treatment), B2: severe bone lesions) for each year (i.e., in 2008 for the initial claim and during 2009 to 2012 for renewal claims), and (3) clinical stage of NF1 (Stage 1: D1 and N0 and B0, Stage 2: (D1 or D2) and (N0 or N1) and (B0 or B1), Stage 3: D3 and N0 and B0, Stage 4: D3 and (N1 or B1), Stage 5: D4 or N2 or B2) for each year. With regard to the D3 category, physicians were permitted to estimate the number of neurofibromas on the entire body based on the number observed on a part of the body. Diagnostic criteria and severity classification of NF1 in Japan, which were developed based on criteria set forth by the National Institutes of Health in 1988 [15], are provided in Table 1 and [see Additional file 1: Appendix 1]. Eligibility criteria for study participants were: (1) new registrants in 2008 and (2) no duplicate data. Data duplication was assessed based on ID number, age (birth date), and sex.

**Table 1:** Participant characteristics by age at registration in 2008

Age	0–19 yrs	20–39 yrs	40–59 yrs	> 60 yrs	Chi-square test
Total	74	109	89	70	
Sex					
Male	32	41	35	40	
Female	42	68	54	30	
Dermatological manifestations ^a)^ (*n* = 340)					*
D1	24	9	4	3	
D2	17	25	13	9	
D3	13	42	47	38	
D4	18	33	25	20	
Neurological manifestations ^b)^ (*n* = 333)					
N0	33	45	37	25	
N1	20	47	33	29	
N2	18	15	16	15	
Bone manifestations ^c)^ (*n* = 331)					*
B0	32	56	46	23	
B1	16	27	30	37	
B2	25	21	10	8	
Clinical stage ^d)^ (*n* = 342)					*
Stage 1, 2	18	24	8	7	
Stage 3, 4	9	29	41	26	
Stage 5	47	56	40	37	

*P < 0.05

a) Dermatological manifestations

D1: Pigmented macules and few neurofibromas

D2: Pigmented macules and many neurofibromas

D3: Numerous neurofibromas (> 1000)

D4: Functional impairment or severe physical distress due to plexiform neurofibromas, or malignant peripheral nerve sheath tumour

b) Neurological manifestations

N0: No neurological symptoms

N1: Neurologic symptoms (e.g., paralysis or pain)

N2: Severe/progressive neurological symptoms

c) Bone manifestations

B0: No bone lesions

B1: Mild/moderate bone lesions (e.g., deformity in spine that does not require surgical treatment)

B2: Severe bone lesions

d) Classification of severity

Stage 1: D1 and N0 and B0

Stage 2: (D1 or D2) and (N0 or N1) and (B0 or B1)

Stage 3: D3 and N0 and B0

Stage 4: D3 and (N1 or B1)

Stage 5: D4 or N2 or B2

Ethical approval was not required based on the “Ethical Guidelines for Medical and Health Research involving Human Subjects” set forth by the Japanese government, as the present study used a completely anonymous database that was provided by the MHLW and was free from any identifiable personal information.

### Statistical analysis

The chi-square test was used to examine whether participant characteristics differed by age at registration in 2008. For this analysis, *P* < 0.05 was considered significant.

Individuals who submitted new claims for medical expense subsidies for NF1 in 2008 were followed until 2012. The observation period was calculated for each participant. Participants who were lost to follow-up were censored at the last confirmed year.

The outcome measure was progression of NF1 clinical stage. Stage progression rates (%) during the follow-up period were calculated using the number of patients who progressed in stage divided by the total number of patients by age and clinical stage at registration. Progression incidence rates were calculated per 100 person-years using the number of outcomes divided by accumulated person-years by age and clinical stage at registration. Because we aimed to examine the stage progression (i.e., worsening of severity) of NF1, stage progression rate and progression incidence rate were calculated for those who were classified as any of Stages 1–4 in 2008.

Of the registrants in 2008, we removed ineligible participants and those with missing data on NF1 severity for statistical analyses. Statistical analyses were performed using SAS version 9.4 (SAS Institute, Cary, NC).

## Results

Of the 357 new registrants in 2008, five were ineligible for inclusion due to duplicate data, and 10 were removed from the analysis due to missing data regarding NF1 severity in 2008. The final study population consisted of 342 participants (194 females and 148 males; mean (SD) age at registration, 39.2 (21.0) years) who newly submitted claims for medical subsidies in 2008 (Fig. 1).

Participant characteristics by age and clinical stage at registration in 2008 are shown in Table 1. More than half of the participants (180/342) were classified as Stage 5 in 2008. Nearly 10% of participants aged 40–59 and > 60 years were classified as Stage 1 or Stage 2 in 2008, while one quarter of those aged 0–19 and 20–39 years were classified as Stage 1 or Stage 2. Mild dermatological manifestations and severe bone manifestations were more prevalent in the 0–19 year age group.

Of the 342 participants, 205 (60%) submitted renewal claims between 2009 and 2012 (Table 2). Over the course of the study period, stage progression was observed in 30 patients, yielding a stage progression rate of 19% and progression incidence rate per 100 person-years of 12.2.

**Table 2:** Stage progression rates and incidence rates of stage progression of NF1 from 2009 to 2012 by age and clinical stage at registration in 2008

	No. of cases (A)	No. of cases with renewal claim (B)	No. of cases with no renewal claims	Renewal rate (B/A)	No. of cases with stage progression (C)	No. of cases with dermatological progression	No. of cases with neurological progression	No. of cases with bone progression	Person-years (D) a)	Stage progression rate (C/A) b)	Incidence rate per 100 person-years (C/D)
Total	342	205	137	60%	30	13	16	8	245.0	19%	12.2
Age group											
0–19 yrs	74	47	27	64%	7	1	4	4	28.0	26%	25.0
20–39 yrs	109	65	44	60%	10	6	7	1	89.0	19%	11.2
40–59 yrs	89	53	36	60%	6	4	2	1	77.5	12%	7.7
> 60 yrs	70	40	30	57%	7	2	3	2	50.5	21%	13.9
Clinical stage											
Stage 1, 2	57	19	38	33%	10	6	7	2	63.0	18%	15.9
Stage 3, 4	105	67	38	64%	20	7	9	6	182.0	19%	11.0
Stage 5	180	119	61	66%	–					–	
Age×clinical stage											
0–19 yrs											
Stage 1, 2	18	7	11	39%	3	0	2	1	20.0	17%	15.0
Stage 3, 4	9	5	4	56%	4	1	2	3	8.0	44%	50.0
Stage 5	47	35	12	74%	–					–	
20–39 yrs											
Stage 1, 2	24	9	15	38%	6	5	4	1	26.5	25%	22.6
Stage 3, 4	29	20	9	69%	4	1	3	0	62.5	14%	6.4
Stage 5	56	36	20	64%	–					–	
40–59 yrs											
Stage 1, 2	8	2	6	25%	1	1	1	0	10.5	13%	9.5
Stage 3, 4	41	23	18	56%	5	3	1	1	67.0	12%	7.5
Stage 5	40	28	12	70%	–					–	
> 60 yrs											
Stage 1, 2	7	1	6	14%	0	–	–	–	6.0	0%	0.0
Stage 3, 4	26	19	7	73%	7	2	3	2	44.5	27%	15.7
Stage 5	37	20	17	54%	–					–	

a) Sum of observation periods of Stage 1 to Stage 4 patients at registration

b) Total number of Stage 1 to Stage 4 patients at registration was used as the denominator to calculate the rate

With regard to age and clinical stage at registration, both stage progression rate and progression incidence rate were the highest in the 0–19 year age group (Table 2). In particular, those who were aged 0–19 years and classified as Stage 3 or Stage 4 in 2008 had the highest stage progression rate and progression incidence rate. Those who were aged 0–19 and 20–39 years and classified as Stage 1 or Stage 2 in 2008 also had high progression incidence rates. However, while progression of neurological and bone manifestations appeared to be more prevalent in the 0–19 year age group, progression of dermatological and neurological manifestations was more prevalent in the 20–39 year age group. Furthermore, manifestations in all of those aged 60 years or above progressed from moderate to severe.

## Discussion

In this study, we examined the progression of NF1 in Japanese patients with NF1 who newly submitted claims for medical expense subsidies in 2008 using national registry data, and followed them until 2012. Both stage progression rate and progression incidence rate were the highest in the 0–19 year age group at the time of registration, and progression of neurological and, in particular, bone manifestations, was more prevalent in this age group as compared to older age groups.

The proportion of participants classified as Stage 1 or Stage 2 at registration in 2008 was higher among those aged 0–19 and 20–39 years than among those aged > 40 years. These characteristics appear to be consistent with a previous clinical study which reported that the grade of NF1 severity tended to be higher in older patients than in younger patients at a university hospital in Japan [7].

The progression of severity for neurological and bone manifestations was more prevalent in the 0–19 year age group than other age groups. This result is consistent with a previous study reporting that bone abnormalities, such as tibial pseudarthrosis and sphenoid wing dysplasia, are typically observed in early infancy [4]. Risk of skeletal abnormalities was also reported to be higher in children aged 16 years or younger [16]. For neurological manifestations, our results are consistent with findings from a clinical study suggesting that children with NF1 are prone to learning disabilities/difficulties, attention deficits, and autism spectrum disorders [17], although mental retardation is rare in these children [4]. Detailed information regarding neurological manifestations was not obtained from the national registry, but most of the neurological manifestations in the 0–19 year age group were likely to be developmental disorders, such as attention deficit and autism spectrum disorders. As with previous studies [3, 18], our findings also suggest that children with NF1 should undergo neuropsychological screening assessments early in life.

The progression of severity of cutaneous and neurological manifestations was more prevalent in the 20–39 year age group than in the 0–19 year age group. This is consistent with previous studies which found that malignant peripheral nerve sheath tumours (MPNST) were present in 8 to 13% of patients with NF1, with a median age of MPNST diagnosis of 26 years [4, 19]. Our findings are also in line with a previous study carried out in Finland using a population-based registry with follow-up data, which estimated the cumulative risk for MPNST in NF1 patients by age 30 years to be 8.5% [12].

Notably, nearly 40% of patients in the present study, who newly submitted claims in 2008, did not apply for renewal during 2009 to 2012. In particular, only 33% of patients who were classified as Stage 1 or Stage 2 in 2008 submitted renewal claims between 2009 and 2012. This could be attributed to the fact that patients with NF1 of mild severity, such as those with mild or moderate dermatological manifestations and no/mild neurological/bone manifestations, were not eligible to receive subsidies.

While international projects using registries of patients with NF1 have been ongoing in western countries [8, 20], the present follow-up study is the first to examine the state of patients with NF1 using a national registry that includes information of all patients with NF1 who submitted claims for medical expense subsidies. As suggested in a previous report [1], national registry systems make it possible to share information about patients with intractable rare diseases among physicians in order to follow their prognosis. The registry we used in the present study is useful for understanding the characteristics of patients with uncommon conditions, such as NF1.

To our knowledge, there has been no study examining the state of NF1 progression using a registry of patients who submitted claims for medical expense subsidies. However, there have been studies regarding other rare diseases, such as amyotrophic lateral sclerosis [21] and spinocerebellar ataxia [1], using a registry of patients who submitted claims to receive medical expense subsidies in Japan. The registration system enables researchers involved in the MHLW research system for intractable rare diseases to utilize the anonymized national registry of individuals with these diseases. The present study suggests that a registry of rare diseases, including NF1, with an administrative purpose is useful for understanding the characteristics, especially prognosis, of patients with uncommon conditions, although the possibility of selection bias should be considered.

This study has several limitations worth noting. First, participants were limited to patients with NF1 who submitted new claims for medical expense subsidies in 2008. Hence, regardless of clinical stage, patients with NF1 who did not apply for subsidies were not included. This could potentially have resulted in selection bias. Second, the follow-up period was relatively short. Unfortunately, information on patients who registered after 2013 was not available for analysis. Finally, participants were limited to Japanese patients. Although previous studies have noted that there are no significant differences in the incidence and prevalence of NF1 across countries and ethnicities [3, 4], caution should be exercised when generalising the present findings to populations with different backgrounds.

## Conclusions

The present study examined the progression of severity of NF1 in Japanese patients who newly submitted claims for medical expense subsidies in 2008 and who could be followed until 2012. Both stage progression rate and progression incidence rate were the highest in the 0–19 year age group at the time of registration and, as compared to other age groups, progression of neurological and bone manifestations was more prevalent in this younger age group. These findings contribute to the understanding of support needs of patients with NF1 by age and clinical stage, the development of evidence-based clinical guidelines, and improvement of the national health care system for NF1. Our findings also highlight the feasibility of conducting quality research using registries of patients with rare diseases, such as NF1, that were not designed specifically for scientific research.

## Additional file

Additional file 1: Appendix 1 Diagnostic criteria of neurofibromatosis type 1 in Japan (DOCX 14 kb)

## Data Availability

The datasets analysed in the current study are not publicly available because the national registry of NF1 was provided specifically for this study by the MHLW.

## Abbreviations

MHLW: Ministry of Health, Labour and Welfare
NF1: Neurofibromatosis type 1
