# Supplementary material for: Evaluation of neurofibromatosis type 1 progression using a nationwide registry of patients who submitted claims for medical expense subsidies in Japan between 2008 and 2012
Source: Orphanet J Rare Dis. 2019 Jul 5;14:166. doi: 10.1186/s13023-019-1148-8 (PMC6612089; doi:10.1186/s13023-019-1148-8)
Supplement: Supplementary file 1 — Appendix 1 Diagnostic criteria of neurofibromatosis type 1 in Japan (DOCX 14 kb) [file 13023_2019_1148_MOESM1_ESM.docx]

Additional file 1

| Appendix 1. Diagnostic criteria of neurofibromatosis type 1 in Japan |
| --- |
| 1. Major symptoms  (1) Café-au-lait macules  (2) Neurofibromas |
| 2. Other symptoms  (1) Dermatological manifestations  (2) Bony manifestations  (3) Eye lesion  (4) Cerebrospinal tumor  (5) Unidentified bright object  (6) Gastrointestinal stromal tumor  (7) Pheochromocytomas  (8) Malignant peripheral nerve sheath tumor  (9) Learning disabilities/attention deficits |
| Diagnosis: 1. Make a diagnosis based on café-au-lait macules and neurofibromas. 2. In pretumorous stage, make a diagnosis based on more than six café-au-lait macules, family history of neurofibromatosis type 1, and other symptoms. 3. In adult cases for which it is difficult to detect cafe-au-lait macules, make a diagnosis based on neurofibromas. |
| Notes: Diagnostic criteria of neurofibromatosis type 1 in Japan were developed based on criteria set forth by the National Institutes of Health in 1988. |
